# Development and psychometric validation of the Physician Occupational Stigma Perception Scale among Chinese physicians

**DOI:** 10.3389/fpubh.2026.1912387

**Published:** 2026-09-11

**Authors:** Yafan Fan, Jun Li, Min Li

**Affiliations:** 1 School of Administration, Hubei University of Chinese Medicine, Wuhan, China; 2 School of Business Administration, South China University of Technology, Guangzhou, China

**Keywords:** medical ethics, medical skills and services, occupational stigma perception, physician, scale development

## Abstract

**Background:**

Occupational stigma is increasingly recognized as an important psychosocial concern for physicians, yet no instrument specifically measures how Chinese physicians perceive negative public evaluations of their profession. This study aimed to develop and psychometrically evaluate the Physician Occupational Stigma Perception Scale (POSPS) for Chinese physicians.

**Methods:**

An exploratory sequential mixed-methods design was used. The POSPS was developed and administered in Chinese. Candidate items were generated from semi-structured interviews with 23 frontline physicians and relevant existing measures, and were subsequently refined through two rounds of Delphi consultation and target-population pretesting. A pilot survey of 193 physicians was used for exploratory factor analysis (EFA), with Horn’s parallel analysis guiding factor retention. The resulting structure was examined in an independent sample of 312 physicians using confirmatory factor analysis (CFA). Internal consistency, convergent and discriminant validity, and nomological validity were also assessed.

**Results:**

EFA yielded a six-item, two-factor solution comprising Medical Ethics Stigma Perception (ME) and Medical Skills and Services Stigma Perception (MSS). The correlated two-factor CFA model showed good fit. Cronbach’s alpha was 0.849 for ME and 0.862 for MSS, and McDonald’s omega was 0.851 and 0.862, respectively. Composite reliability values were 0.852 and 0.866, and average variance extracted values were 0.659 and 0.684. The latent correlation between ME and MSS was 0.496, supporting their related but distinct nature. Both dimensions were positively correlated with emotional exhaustion and ambivalent occupational identification and negatively correlated with job satisfaction, providing evidence of nomological validity.

**Conclusion:**

The six-item POSPS demonstrated acceptable psychometric properties for assessing occupational stigma perceptions among Chinese physicians. Its two subscales provide a concise, contextually grounded measure for research on physicians’ occupational experiences and well-being in China.

## 1 Introduction

The performance of a public health system depends not only on its financing and institutions but also on the physicians who deliver care at its front line. In China, current health care reform emphasizes coordination among health services, health insurance, and pharmaceutical management, commonly described as three-medical synergy. This reform forms part of a broader shift from treatment-centered care to health-centered governance (1). Because each pillar is enacted through physicians’ everyday decisions, their professional standing is itself a public health resource; yet occupational stigma has become a growing problem for the profession, and the perception of stigma in healthcare settings can undermine the success of diagnosis and treatment (2). Characterized as either “angels in white” or “wolves in white (3),” physicians increasingly work under a stigmatized image: in the 2021 Chinese Physician Survey Report, more than 65% reported feeling that the public misunderstands physicians and that the pressure of public opinion is great, with the misconduct of a few extended to the whole group (4). Physicians’ occupational stigma is also a global issue: during the COVID-19 pandemic, physicians were labeled “plague bearers,” ostracized, and subjected to verbal and physical attacks (5), and the International Committee of the Red Cross recorded over 600 incidents of violence and harassment against healthcare workers and facilities within 6 months of the outbreak (6).

Physicians’ occupational stigma perception is not merely an individual psychological burden. It may also have broader implications for physicians’ occupational well-being, professional attitudes, and the delivery of healthcare services. Public suspicion that physicians profit from over-examination, over-prescription, and over-treatment can reinforce a stigmatizing image of the profession. At the same time, routine constraints in clinical practice, such as long waiting times, brief consultations, and uncertainty in treatment outcomes, may be interpreted by patients as evidence of inadequate competence, indifference, or misconduct, thereby contributing to negative public stereotypes of physicians (3, 4). Perceived stigma has been associated with adverse occupational outcomes, including emotional exhaustion, conflicted occupational identification, and lower job satisfaction (6). Accurately assessing how physicians perceive such negative public evaluations is therefore important for understanding an underrecognized psychosocial aspect of physicians’ working environment and for informing efforts to support the medical workforce.

Occupational stigma perception refers to an individual’s awareness and experience of the negative evaluations, labels, stereotypes, prejudices, and discrimination associated with their profession (7). Despite increasing attention to occupational stigma, its conceptualization and measurement remain heterogeneous across occupations, and existing instruments do not adequately capture how Chinese physicians perceive stigma directed toward their profession (4). The most widely used instruments are the Stigma Consciousness Questionnaire (SCQ) and the dirty-work Occupational Stigma Perception Scale adapted from Schaubroeck (4, 8); however, both were developed in Western contexts and were not designed specifically around the social evaluations encountered by physicians in the Chinese healthcare setting. Moreover, much of the occupational-stigma literature has focused on occupations traditionally characterized as “dirty work,” whereas physicians occupy a socially prestigious but increasingly contested professional position; consequently, measures developed for dirty-work occupations may not fully capture the content of stigma perceived by physicians (3, 7, 9). Within healthcare, several related scales exist, including the Forensic Patient Stigma Scale (10), the Patient Toward Physician Occupational Stigma Scale (11), the Nurse Occupational Stigma Scale (12), and the Physician Internalized Occupational Stigma Scale (PIOSS), but none directly measures physicians’ own perception of occupational stigma. The PIOSS, in particular, captures the extent to which physicians identify with and internalize the public’s negative labels as self-concepts, which is conceptually distinct from perceiving negative public evaluations of the medical profession. Thus, a brief physician-specific instrument for assessing perceived occupational stigma remains lacking (11, 13, 14).

Scholars have further called for occupational stigma perception to be studied across different cultures (7, 8). The meanings attached to occupational stigma are shaped by social expectations, professional norms, and the institutional context in which an occupation is practiced. The public image of Chinese physicians has undergone substantial change alongside transformations in the healthcare system and physician - patient relationships. Although physicians have traditionally occupied a respected professional position, concerns surrounding financial incentives, medical disputes, service expectations, and episodes of violence against healthcare workers have contributed to increasingly ambivalent and, at times, negative social evaluations of the profession (3). These context-specific features suggest that simply transferring an occupational stigma instrument developed for another profession or cultural setting may omit important aspects of how Chinese physicians experience public devaluation. A measure grounded in physicians’ own accounts and the Chinese healthcare context is therefore needed.

Therefore, the present study aimed to develop and psychometrically evaluate a Physician Occupational Stigma Perception Scale (POSPS) specifically for Chinese physicians. Using an exploratory sequential mixed-methods design, candidate items were first generated from qualitative interviews and relevant existing measures and were subsequently refined through two rounds of expert consultation and target-population pretesting. The preliminary scale was then examined using EFA, including parallel analysis to guide factor retention, and the resulting structure was evaluated in an independent physician sample using CFA. Internal consistency, convergent validity, discriminant validity, and nomological validity were further assessed. By providing a concise and contextually grounded measure of physicians’ perceived occupational stigma, the POSPS is intended to facilitate research on the social and occupational experiences of Chinese physicians and their associations with occupational well-being.

## 2 Theoretical background and conceptualization

### 2.1 Theoretical foundations

This study adopted Corrigan et al.’s social-cognitive model of public stigma as the primary framework for conceptualizing physicians’ occupational stigma perception. The model describes public stigma as a social-cognitive process involving social cues or labels, stereotypes, prejudice, and discrimination (15, 16). Once an individual is identified as a member of a socially meaningful group, culturally shared stereotypes may be activated. When these stereotypes are endorsed, they may generate prejudicial judgments and negative emotional reactions, which can subsequently lead to discriminatory intentions or behaviors. Identification as a physician may activate public beliefs about physicians’ morality, financial motives, competence, service attitudes, or use of professional authority. Link and Phelan’s conceptualization of stigma provided a complementary perspective by emphasizing labeling, stereotyping, separation, status loss, and discrimination within relations of social power (17).

The social-cognitive framework provided a theoretical basis for organizing the qualitative item-development process. The semistructured interviews were designed to elicit the negative labels, stereotypes, prejudices, and discriminatory experiences that physicians perceived to be directed at their occupation. Participants were therefore asked what stigma, prejudice, discrimination, stereotypes, or other negative labels physicians encountered compared with other occupations and were encouraged to provide specific examples. During coding and item generation, interview material was considered relevant when it reflected an externally attributed negative evaluation of physicians as an occupational group. Candidate items were formulated to represent identifiable public, patient, or family stereotypes and prejudicial judgments, whereas content reflecting physicians’ own opinions, internalized stigma, purely factual misunderstandings, or consequences of stigma was excluded.

The framework also informed expectations regarding dimensionality without imposing a predetermined factor structure. Because stigmatizing evaluations may target different aspects of physicians’ occupational identity, occupational stigma perception was expected to contain potentially distinguishable content domains rather than constitute a single undifferentiated construct. However, the theoretical framework did not prescribe the number or labels of these dimensions. The provisional content domains were derived from qualitative analysis, and their empirical organization was subsequently examined through EFA and CFA. Thus, the theoretical framework defined the boundaries of occupational stigma, the interviews identified its occupation-specific content, and factor analysis determined how that content was organized psychometrically.

### 2.2 Construct definition and boundary

In this study, physicians’ occupational stigma perception was defined as physicians’ awareness and experience of negative stereotypes, prejudicial judgments, and discriminatory expectations directed at the medical profession by members of the public, including patients and their families. The construct therefore focuses on perceived external stigma, that is, how physicians perceive relevant external audiences to view and evaluate their profession. Three features define the construct. First, the stigmatizing evaluation has an external referent, such as the public, patients, or patients’ families. Second, it contains negative or devaluing content rather than a neutral description of the occupation (7). Third, the evaluation must be attributed to membership in the medical profession rather than solely to the individual conduct of a particular physician.

This definition distinguishes physicians’ occupational stigma perception from several related constructs. Physicians’ occupational stigma perception is distinct from generalized stigma consciousness, which refers to a broader expectation that one will be stereotyped because of group membership (18). Although generalized stigma-consciousness items were considered during item-pool development, the present construct focuses on the specific negative beliefs that physicians perceive to be directed at medical ethics, clinical competence, and service behavior. The construct must also be distinguished from internalized occupational stigma. Internalization occurs when physicians accept negative public stereotypes and incorporate them into their own self-concept (4). By contrast, physicians may perceive that the public holds stigmatizing beliefs without personally endorsing or internalizing them. Measures of physician internalized occupational stigma assess a theoretically distinct construct from perceived external occupational stigma (4). Accordingly, the POSPS was designed to assess physicians’ perceptions of occupation-specific external stereotypes and prejudicial evaluations.

## 3 Materials and methods

### 3.1 Methodology

#### 3.1.1 Design

**Figure 1:**
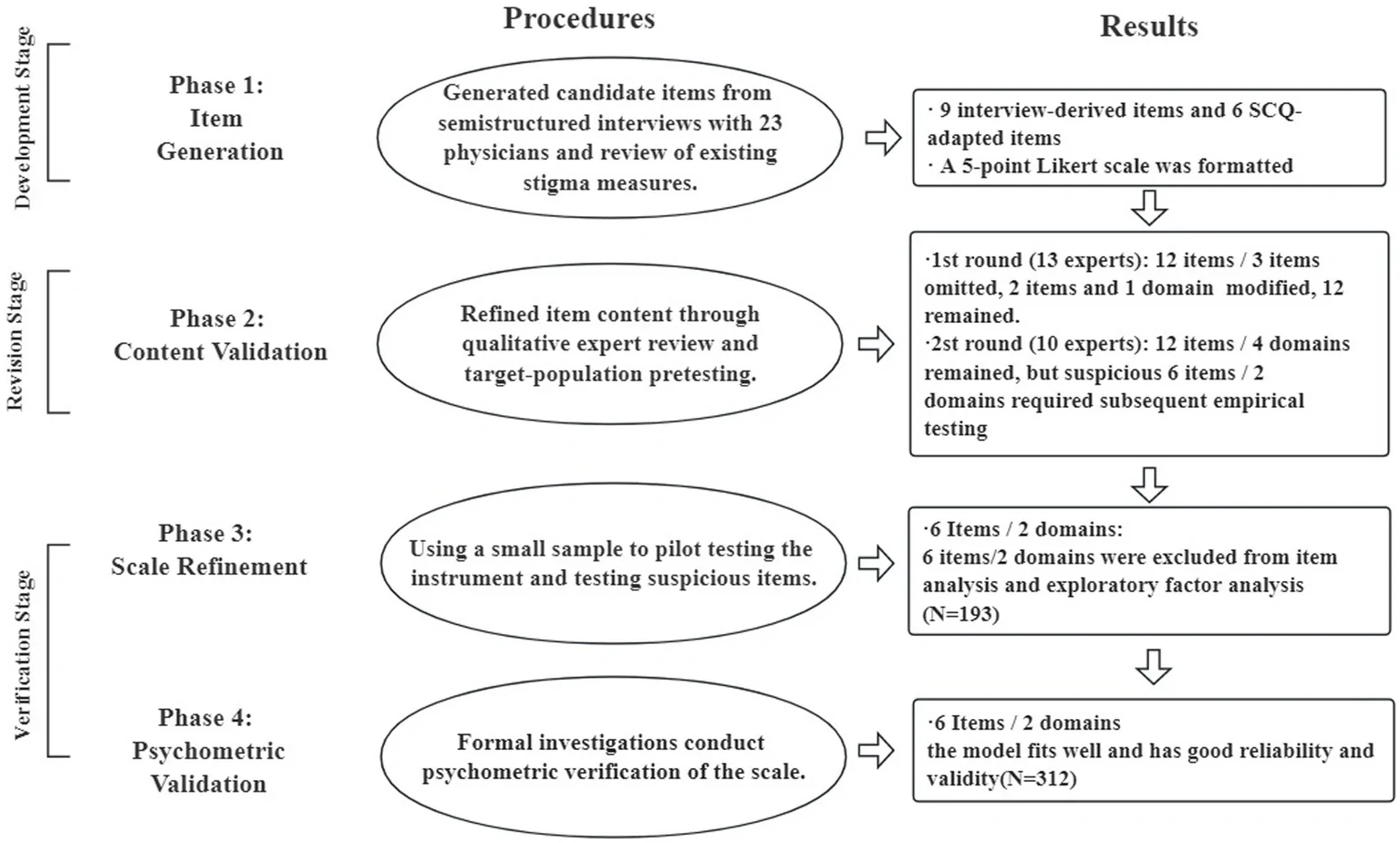
Xxx.

An exploratory sequential mixed-methods design was employed to develop and validate the POSPS (19). The process consisted of four sequential phases: item generation, content validation and item pretesting, scale refinement, and psychometric validation (Figure 1). The qualitative phase was used to identify occupation-specific stigma content and generate candidate items, which were subsequently evaluated and refined through quantitative analyses. Phase 3 used an exploratory sample of 193 physicians to examine the dimensional structure of the preliminary scale, whereas Phase 4 used an independent sample of 312 physicians to confirm the factor structure and evaluate the psychometric properties of the final POSPS.

#### 3.1.2 Item generation

The initial item pool was developed from two complementary sources: semistructured interviews with physicians and a review of existing stigma measures. The complete interview guide is provided in Supplementary file 1. Interviews were conducted individually by video meeting, telephone, or face to face. The semistructured guide consisted primarily of open - ended questions and follow-up probes; no closed-ended questions were used for item generation. Interviews lasted 30–90 min (mean = 46 min, total = 1,058 min). With participant consent, interviews were audio - recorded when feasible; otherwise, detailed contemporaneous notes were taken. Recordings and notes were transcribed and expanded into 109,797 Chinese characters. Eighteen physicians were interviewed initially, followed by five additional interviews; the additional interviews yielded no new major category or candidate-item concept, and conceptual saturation was therefore judged to have been reached. Theoretical sampling was used to recruit frontline clinical physicians because of their frequent and direct interactions with patients and their families. The final qualitative sample comprised 23 frontline physicians from six tertiary public hospitals in Beijing, Wuhan, and Guangzhou, representing northern, central, and southern China, respectively. The qualitative phase was designed to capture the core manifestations of physicians’ occupational stigma perception. Two doctoral researchers independently analyzed the interview data using a three-level coding procedure consisting of open, axial, and selective coding. Coding continued until no new major concepts emerged. The qualitative findings were translated into candidate items and supplemented by a review of existing stigma measures. Six items adapted from the Stigma Consciousness Questionnaire were included to examine the applicability of generalized stigma-consciousness content to physicians. Together, the interview-derived and adapted items formed the candidate item pool for subsequent content validation and item pretesting.

#### 3.1.3 Content validation and item pretesting

Content validity was evaluated through two rounds of Delphi expert consultation. In the first round, 13 experts with relevant academic or professional experience evaluated the 15 candidate items. The consultation questionnaire included information on the study background and construct definition, item-importance ratings, open-ended comments, and questions concerning the experts’ familiarity with the topic and the basis of their judgments. Each item was rated on a five-point scale ranging from 1 (very unimportant) to 5 (very important). Ten experts participated in the second round and re-evaluated the revised 12-item pool.

The quality of the consultation was assessed using the expert familiarity coefficient (Cs), judgment coefficient (Ca), authority coefficient (Cr), mean importance score (Mj), coefficient of variation (CV), full-score rate, and Kendall’s coefficient of concordance (W). Items with an Mj below the overall mean minus one standard deviation, a CV above the overall mean plus one standard deviation, or a full-score rate below 50% were flagged for further review. These criteria were not applied as automatic deletion rules. Final decisions regarding item retention, revision, or removal also considered experts’ qualitative comments, conceptual relevance, occupation specificity, and content coverage.

After the Delphi consultation, the revised items were individually reviewed by 10 frontline physicians from the target population using a structured item-pretesting form. The review focused on item clarity, comprehension, wording, response format, interpretability, and whether each item was understood as reflecting perceived evaluations from external audiences rather than respondents’ own opinions. Participants provided qualitative feedback on each item, and no quantitative face-validity index was calculated. Based on this feedback, minor wording and presentation refinements were made where necessary, and a five-point Likert response format ranging from 1 (strongly disagree) to 5 (strongly agree) was finalized.

#### 3.1.4 Scale refinement

The third phase involved a pilot survey to refine the preliminary POSPS and examine its latent structure. Recruitment was conducted across tertiary public hospitals in eastern, central, and western China to enhance geographic variation while maintaining relative homogeneity in institutional level. Physicians who had participated in the preceding qualitative interviews assisted in establishing contact with their hospitals, and questionnaire distribution was further facilitated by hospital human-resources departments. Eligible frontline physicians were subsequently recruited using a referral-assisted snowball approach. A total of 200 questionnaires were distributed. Before completing the questionnaire, all participants were informed of the study purpose, the voluntary nature of participation, and the confidentiality and anonymity of their responses, and informed consent was obtained from each participant. After questionnaires with logical inconsistencies, uniform response patterns, or more than 10% missing data were excluded, 193 valid responses remained, yielding an effective response rate of 96.50%. The sample size exceeded the commonly recommended minimum of five participants per item for EFA; with 12 preliminary items, the final sample provided approximately 16 participants per item. Participants responded to each item using a five-point Likert scale ranging from 1 (strongly disagree) to 5 (strongly agree).

Before EFA, the data were screened for missing values, item-response distributions, skewness and kurtosis, floor and ceiling effects, and univariate and multivariate outliers. Potential univariate and multivariate outliers were identified using standardized scores and Mahalanobis distance, respectively, and their influence on the factor solution was examined through sensitivity analysis rather than by automatic case deletion. All 12 preliminary items were entered into the initial EFA before any reliability-based item deletion. Principal axis factoring with Promax rotation was used because the underlying factors were expected to be correlated. Factor retention was determined using the scree plot, observed eigenvalues, Horn’s parallel analysis, and theoretical interpretability. Item refinement was iterative, considering communalities, primary loadings, cross-loadings, conceptual coherence, and adequate factor representation. Items were removed one at a time, and the factor-retention decision and EFA solution were re-evaluated after each removal.

#### 3.1.5 Psychometric validation

The fourth phase used an independent physician sample, with no overlap with the scale-refinement sample, to confirm the factor structure and evaluate the psychometric properties of the six-item POSPS. Participants were frontline physicians recruited from public tertiary hospitals. The same data-quality criteria used in the scale-refinement phase were applied, and 312 valid questionnaires were retained for analysis. Participant characteristics are presented in Supplementary file 15. Before completing the questionnaire, participants were informed of the study purpose, the voluntary nature of participation, and the confidentiality and anonymity of their responses, and informed consent was obtained.

The formal survey included the six-item POSPS and three theoretically relevant external measures. The POSPS comprised two three-item subscales, ME and MSS. Each item was rated on a five-point Likert scale ranging from 1 (strongly disagree) to 5 (strongly agree), with higher subscale scores indicating greater perceived occupational stigma in the corresponding domain. ME and MSS were scored and interpreted separately; an overall POSPS score was not prespecified for interpretation because the formal validation was intended to evaluate the two-dimensional structure identified in the exploratory phase.

Emotional exhaustion was assessed using the three-item measure developed by Boswell et al. (20), ambivalent occupational identification using the three-item measure developed by Ashforth et al. (21), and job satisfaction using the five-item measure developed by Judge et al. (22). All three measures used five-point response formats. These constructs were selected to situate the POSPS within a theoretically relevant nomological network. Based on previous occupational-stigma research, both POSPS dimensions were expected to correlate positively with emotional exhaustion and ambivalent occupational identification and negatively with job satisfaction. Psychometric evaluation of the formal-survey data included confirmatory factor analysis, internal-consistency reliability, convergent and discriminant validity, and nomological validity; the corresponding statistical procedures are described in Section 3.2.

### 3.2 Data analysis

Data analysis proceeded sequentially in accordance with the exploratory mixed-methods design and the phases of scale development and psychometric validation. Qualitative interview data were managed and analyzed using MAXQDA 2022. Two researchers independently coded the interview transcripts through open, axial, and selective coding. Initial concepts were identified through line-by-line coding and subsequently compared, merged, and organized into higher-order categories. Disagreements were resolved through discussion and, when necessary, consultation with a third researcher. Intercoder agreement was assessed using Cohen’s kappa. Data collection and coding continued until no substantively new categories emerged (23). The two rounds of Delphi expert consultation were evaluated using the Ca, Cs, Cr, Mj, CV, full-score rate, and W (24). The authority coefficient was calculated as Cr = (Ca + Cs)/2, with values of 0.70 or above considered indicative of acceptable expert authority (25). The concentration of expert opinions was assessed using Mj and CV, while Kendall’s W and its associated chi-square test were used to assess agreement among experts. Item decisions were not based on a single numerical rule. Quantitative ratings were considered together with experts’ qualitative comments regarding conceptual relevance, physician specificity, comprehensibility, and cultural appropriateness.

Scale refinement was conducted using the pilot-survey data in SPSS26.0, Amos 24.0, and Mplus 8.3. Before EFA, item distributions, missing data, skewness, kurtosis, floor and ceiling effects, and potential univariate and multivariate outliers were examined. Standardized scores with an absolute value greater than 3.29 were used to flag potential univariate outliers, and Mahalanobis distance was used to identify potential multivariate outliers at *p* < 0.001. Multivariate normality was additionally assessed using Mardia’s multivariate kurtosis statistic in Amos 24.0. Potential outlying observations were not removed solely on statistical grounds; sensitivity analyses were conducted where appropriate to determine whether their exclusion materially affected the factor solution (24). All 12 preliminary POSPS items were entered into the initial EFA before any item was considered for deletion. EFA was performed in SPSS 26.0 using principal axis factoring with Promax oblique rotation, because correlations among the underlying dimensions were theoretically plausible. Sampling adequacy and factorability were assessed using the Kaiser - Meyer - Olkin (KMO) measure and Bartlett’s test of sphericity. Factor retention was determined from several sources of evidence rather than from the eigenvalue-greater-than-one criterion alone. Specifically, the scree plot, theoretical interpretability, and Horn’s parallel analysis were jointly considered (26). Parallel analysis was conducted in Mplus 8.3 using 1,000 randomly generated datasets, with observed eigenvalues compared with the corresponding 95th-percentile random eigenvalues (27).

Item refinement was iterative. Pattern coefficients below |0.30| were suppressed for presentation, while loadings of approximately 0.40 or greater were regarded as desirable for substantive interpretation. Communalities, the magnitude and specificity of primary loadings, cross-loadings, conceptual consistency with the emerging factor, and contribution to construct coverage were considered jointly when evaluating individual items. No item was deleted solely because it failed to meet a single numerical cutoff. Following each item deletion, the EFA and factor-retention analyses were repeated until a statistically interpretable and conceptually coherent solution was obtained. This approach is consistent with recommendations that scale refinement should integrate statistical evidence with theoretical and content considerations rather than rely on mechanical cutoff rules (27, 28).

After the factor structure had been established, internal consistency of the refined subscales was evaluated using Cronbach’s alpha and McDonald’s omega. Coefficients of 0.70 or above were considered acceptable, whereas values of 0.80 or above were interpreted as indicating good internal consistency (29). McDonald’s omega was estimated in SPSS 26.0 using the procedure described by Hayes and Coutts.

CFA was conducted on the independent formal-survey sample using Mplus 8.3. The six POSPS items were treated as approximately continuous indicators and estimated using maximum likelihood estimation with robust standard errors (MLR) (30). Bootstrapping was not used for the CFA. Potential departures from multivariate normality were addressed using the MLR estimator, which provides robust standard errors and a robust model chi-square statistic. The hypothesized correlated two-factor model was compared with a competing single-factor model. For model identification, the first unstandardized factor loading of each latent factor was fixed at 1.0, while the latent variances and the covariance between ME and MSS were freely estimated. Model fit was evaluated using the chi-square statistic (χ2), degrees of freedom, χ2/df, comparative fit index (CFI), Tucker - Lewis index (TLI), root mean square error of approximation (RMSEA), and standardized root mean square residual (SRMR). CFI and TLI values of 0.90 or higher and RMSEA values below 0.08 were regarded as indicative of acceptable fit, whereas values approaching CFI/TLI ≥ 0.95 and RMSEA ≤ 0.06 indicated more stringent evidence of good fit; an SRMR of 0.08 or below was considered acceptable (24). These indices were interpreted jointly rather than as rigid pass - fail criteria. The Akaike information criterion (AIC) and Bayesian information criterion (BIC) were additionally used to compare the competing models, with lower values indicating better relative fit.

Standardized factor loadings, standard errors, residual variances, and the latent correlation between the two POSPS factors were examined to characterize the final measurement model. Modification indices were inspected using a minimum printing threshold of 10. No modification index exceeded this threshold, and no post-hoc correlated residuals or cross-loadings were added. Internal consistency in the formal-survey sample was evaluated separately for the two POSPS subscales using Cronbach’s alpha and McDonald’s omega. Composite reliability (CR) and average variance extracted (AVE) were calculated to evaluate convergent validity. CR values of 0.70 or higher and AVE values of 0.50 or higher were considered acceptable. Discriminant validity was assessed using the Fornell - Larcker criterion, whereby the square root of the AVE for each factor should exceed the correlation between the latent factors (31).

Nomological validity was examined using two-tailed Pearson correlation coefficients between the two POSPS subscales and theoretically relevant external variables, including emotional exhaustion, ambivalent occupational identification, and job satisfaction. Associations in the theoretically expected directions were interpreted as supporting nomological validity. Because the data were cross-sectional, these relationships were interpreted as associations rather than causal effects. All statistical tests were two-tailed, with *p* < 0.05 considered statistically significant.

## 4 Results

### 4.1 Initial item generation

The initial interview-derived item pool was developed from 23 semistructured interviews. Participant characteristics are presented in Supplementary file 2. The interview data were independently coded by two researchers using MAXQDA 2022. Intercoder agreement was assessed using the Kappa coefficient and was 0.80, indicating substantial agreement between the two coders (32). Coding discrepancies were subsequently discussed and resolved by consensus.

Following open, axial, and selective coding, 34 initial concepts were identified. After synonymous expressions were consolidated and single-occurrence codes were removed according to the original coding protocol, 29 concepts remained. These concepts were organized into 11 subcategories and four provisional content domains: social stigma perception, moral stigma perception, medical skills and services stigma perception, and misunderstanding. The detailed coding process is presented in Supplementary file 3. The misunderstanding domain was excluded because its content did not consistently represent negative labeling, stereotyping, prejudice, or discrimination.

Based on the remaining qualitative findings, nine interview-derived candidate items were developed. Six items adapted from the Stigma Consciousness Questionnaire were added to broaden the item pool. The draft items were refined through research-team discussion to improve clarity, conceptual consistency, and cultural appropriateness. This process resulted in an initial pool of 15 candidate items (Supplementary file 4).

### 4.2 Item revision and content validation of the POSPS

#### 4.2.1 Findings from the first round of Delphi expert consultation

A total of 13 experts completed the first round of Delphi consultation. Characteristics of the expert panel are presented in Supplementary file 5. The Cr was 0.88, based on the Ca of 0.90 and the Cs of 0.85, indicating a high level of expert authority.

Across the 15 candidate items, the Mj was 4.09, and the CVs ranged from 0.00 to 0.21. Detailed item-level results are presented in Supplementary file 6. W was 0.84 (χ^2^ = 153.63, df = 14, *p* < 0.001), indicating a high level of agreement among the experts.

Based on the quantitative ratings and qualitative comments, items C11, C13, and C14 were removed. These three items received the lowest importance ratings (M = 2.46, 2.85, and 2.85, respectively), and none of the experts assigned them the maximum importance score. Their wording was also considered too general or insufficiently specific to physicians’ occupational stigma. In particular, the statements “My occupation affects how others perceive me” and “When communicating with people outside our profession, I feel that they view and interpret my behavior on the basis of my occupation” were considered ambiguous when transferred from a generalized stigma-consciousness context to the physician occupation. Several retained items were revised for clarity and contextual relevance. For example, “People believe that older physicians are more professional” was reframed to describe prejudice toward younger and less experienced physicians, while the gender-related item was revised to describe gender-based discrimination more explicitly. The wording “Many people outside our profession find it difficult to view us equally” was changed to “Many people outside our profession find it difficult to view us objectively.” The provisional domain label “moral stigma perception” was also revised to “medical ethics stigma perception” to better reflect the physician context. The perceptual frame of the retained items was also clarified so that responses referred explicitly to physicians’ perceptions of external evaluations rather than to respondents’ own endorsement of those evaluations. In the English presentation of the revised items, this distinction is made explicit using formulations such as “I perceive that members of the public believe…” and “I perceive that patients and their families…”. Following the deletion of C11, C13, and C14 and the semantic refinement of the retained items, the candidate pool was reduced from 15 to 12 items. A detailed comparison between the original and current item codes is presented in Supplementary file 7.

#### 4.2.2 Findings from the second round of Delphi expert consultation

The second Delphi round was completed by 10 of the 13 original experts (effective response rate = 76.92%). Expert authority remained high (Cr = 0.88). The Mj increased to 4.48, while item-level CVs ranged from 0.00 to 0.17. W was 0.77 (χ^2^ = 84.23, df = 11, *p* < 0.001), indicating a high level of agreement among the experts. Detailed item-level results are presented in Supplementary file 7.

The medical ethics and medical skills and services items received particularly high importance ratings, with mean scores ranging from 4.90 to 5.00. In contrast, items concerning social stigma and generalized stigma consciousness received comparatively lower ratings, ranging from 3.80 to 4.20. Experts noted that generalized stigma-consciousness items were broader and less specific to physicians, whereas seniority-, gender-, and infection-related stigma could be more context-dependent because physicians’ exposure to these experiences may vary across demographic and clinical settings. Nevertheless, the panel considered these contents potentially meaningful and recommended retaining them for subsequent empirical testing rather than excluding them solely on the basis of expert ratings.

Accordingly, no additional items were removed during the second round. All 12 revised items were retained and renumbered as OS1 - OS12 for target-population item pretesting and subsequent psychometric evaluation.

### 4.3 Pilot testing and scale refinement of the POSPS

#### 4.3.1 Preliminary item and data screening

Of the 200 questionnaires distributed, 193 met the prespecified data-quality criteria and were retained for analysis, yielding an effective response rate of 96.50%. The seven excluded questionnaires contained logical inconsistencies, uniform response patterns, or more than 10% missing data. All 193 retained questionnaires were complete, with no item-level missing values. Their demographic characteristics are presented in Supplementary file 8.

Descriptive statistics for the 12 preliminary POSPS items are presented in Supplementary file 9. Item means ranged from 2.03 to 4.24, with standard deviations ranging from 0.801 to 1.283. Skewness ranged from −0.948 to 1.012 and kurtosis from −1.498 to 0.953, indicating no severe univariate distributional departures. Multivariate normality was further assessed using Mardia’s multivariate kurtosis statistic. The multivariate kurtosis value was −2.591 (c.r. = − 0.982), indicating no significant departure from multivariate normality.

Floor effects ranged from 0.0 to 33.2%, whereas ceiling effects ranged from 2.6 to 49.2%. The most pronounced floor concentration was observed for OS9 (33.2%), whereas the largest ceiling concentrations were observed for OS6 (49.2%) and OS5 (42.0%). These distributional characteristics were documented but were not used as stand-alone criteria for item removal.

Univariate outliers were screened using standardized scores with a criterion of |z| > 3.29. Six item-level observations across five participants met this criterion, involving OS3, OS4, and OS6. All represented valid responses within the prespecified five-point response range and were therefore retained. Multivariate outliers were assessed using Mahalanobis distance. One participant exceeded the χ^2^ criterion for 12 variables at *p* < 0.001 (D^2^ = 37.187; critical value = 32.91). A sensitivity analysis excluding this participant produced the same factor-retention decisions and substantively unchanged EFA solutions for both the initial 12 - item pool and the final six-item scale. Detailed results of the sensitivity analysis are presented in Supplementary file 10. Because there was no independent evidence of an invalid response pattern, the participant was retained in the primary analyses. Accordingly, all subsequent scale-refinement analyses were based on the full sample of 193 physicians.

#### 4.3.2 Exploratory factor analysis

All 12 preliminary POSPS items were included in the initial EFA. The KMO measure was 0.803, and Bartlett’s test of sphericity was significant, χ^2^(66) = 772.262, *p* < 0.001, indicating that the correlation matrix was suitable for factor analysis. Principal axis factoring with Promax rotation was used. The initial correlation matrix yielded five eigenvalues greater than 1.0 (4.031, 1.508, 1.373, 1.106, and 1.005). However, the Kaiser criterion was not used as the sole basis for determining the number of factors. The scree plot was inspected as an initial guide, and Horn’s parallel analysis provided a more objective criterion for factor retention. Parallel analysis was conducted in Mplus using 1,000 random datasets, with observed eigenvalues compared against the corresponding 95th-percentile random eigenvalues. The first three observed eigenvalues (4.031, 1.508, and 1.373) exceeded their corresponding random values (1.545, 1.391, and 1.290), whereas the fourth and fifth observed eigenvalues (1.106 and 1.005) fell below the corresponding random values (1.201 and 1.129). Taken together, these results supported retention of three factors for the initial EFA.

Item refinement proceeded iteratively, with one item removed at a time and the factor-retention decision re-examined after each deletion. In the initial 12-item solution, OS12 had a communality of 0.030 and no pattern coefficient of |0.30| or greater and was removed first. Parallel analysis continued to support three factors after removal of OS12. OS7 was then removed because of its low communality (h^2^ = 0.164) and weak primary loading (0.309). In the subsequent 10-item solution, OS8 showed a low communality (h^2^ = 0.264), a weak primary loading (0.354), and poor conceptual alignment with the factor on which it loaded; OS11, although also showing a low communality, retained a more interpretable loading on the provisional third factor. OS8 was therefore removed. Parallel analysis of the resulting nine-item set continued to support three factors. OS11 was removed at the next iteration because its communality was only 0.170. After removal of OS11, parallel analysis of the remaining eight items supported two rather than three factors: the first two observed eigenvalues (3.618 and 1.389) exceeded the corresponding 95th-percentile random eigenvalues (1.409 and 1.268), whereas the third observed eigenvalue (1.007) did not exceed the random value (1.162). The eight items were therefore re-estimated under a two-factor solution. OS10 then showed an extremely low communality (h^2^ = 0.057) and no salient loading of |0.30| or greater on either retained factor and was removed. Parallel analysis of the remaining seven items continued to support two factors. OS9 was subsequently removed because it had a low communality (h^2^ = 0.241), a weak loading of 0.349, and did not align conceptually with the medical ethics factor on which it loaded. The complete sequence of factor-retention results and item-refinement decisions is reported in Supplementary files 11–14. Because the decision to remove OS8 involved both weak factorial performance and conceptual inconsistency, the EFA solutions immediately before and after its removal are provided in Supplementary file 14 to document the stability of the emerging factor structure.

The final six-item set was subjected to a new parallel analysis and EFA. Parallel analysis supported two factors: the first two observed eigenvalues (3.280 and 1.372) exceeded the corresponding 95th-percentile random eigenvalues (1.343 and 1.196), whereas the third observed eigenvalue (0.468) was below the random value (1.088). The final correlation matrix remained suitable for factor analysis (KMO = 0.780; Bartlett’s χ^2^(15) = 557.609, *p* < 0.001). The two-factor solution accounted for 66.67% of the total variance. OS1-OS3 loaded on the first factor, Medical Ethics Stigma Perception (ME), with pattern coefficients ranging from 0.738 to 0.889. OS4-OS6 loaded on the second factor, Medical Skills and Services Stigma Perception (MSS), with coefficients ranging from 0.733 to 0.904. Communalities ranged from 0.588 to 0.793, and no cross-loading of |0.30| or greater was observed. The two factors were moderately correlated (r = 0.470). The final EFA results are presented in Table 1.

**Table 1:** Exploratory factor analysis of the final six-item POSPS (*N* = 193).

Item	Medical Ethics Stigma Perception (ME)	Medical Skills and Services Stigma perception (MSS)	Communality (h^2^)
OS1	0.889	—	0.793
OS2	0.738	—	0.602
OS3	0.811	—	0.615
OS4	—	0.904	0.786
OS5	—	0.784	0.588
OS6	—	0.733	0.615

Principal axis factoring with Promax rotation was used. Pattern coefficients with absolute values below 0.30 were suppressed. KMO = 0.780; Bartlett’s χ^2^ (15) = 557.609, *p* < 0.001; cumulative variance explained = 66.67%; factor correlation = 0.470. POSPS, Physician Occupational Stigma Perception Scale.

#### 4.3.3 Reliability of the refined POSPS

Internal consistency was assessed after the dimensional structure of the POSPS had been established, with reliability evaluated separately for the two EFA-derived subscales (Table 2). The three-item Medical Ethics Stigma Perception (ME) subscale showed good internal consistency, with a Cronbach’s α of 0.854 and a McDonald’s ω of 0.857. Corrected item - total correlations ranged from 0.701 to 0.777, and Cronbach’s α values after deletion of individual items ranged from 0.747 to 0.821. The three-item Medical Skills and Services Stigma Perception (MSS) subscale likewise demonstrated good internal consistency (Cronbach’s α = 0.848; McDonald’s ω = 0.849). Its corrected item - total correlations ranged from 0.687 to 0.768, while α values after deletion of individual items ranged from 0.744 to 0.818. For both subscales, deletion of any single item did not improve internal consistency beyond the corresponding full-subscale α, supporting retention of all six items in the refined POSPS.

**Table 2:** Internal consistency of the refined POSPS subscales (*N* = 193).

Subscale	Item	Mean	SD	CITC	Cronbach’s α if item deleted	Cronbach’s α	McDonald’s ω	Mean inter-item correlation
Medical Ethics Stigma Perception (ME)	OS1	3.97	0.910	0.777	0.747	0.854	0.857	0.662
	OS2	4.06	0.899	0.702	0.819			
	OS3	4.00	0.941	0.701	0.821			
Medical skills and services stigma perception (MSS)	OS4	4.06	0.801	0.768	0.744	0.848	0.849	0.654
	OS5	4.10	0.863	0.687	0.818			
	OS6	4.24	0.881	0.701	0.806			

CITC, corrected item - total correlation; SD, standard deviation.

### 4.4 Formal survey and psychometric validation of the POSPS

#### 4.4.1 Confirmatory factor analysis

Of the 330 questionnaires distributed, 312 met the prespecified data-quality criteria and were retained for analysis, yielding an effective response rate of 94.55%. The 18 excluded questionnaires contained logical inconsistencies, uniform response patterns, or more than 10% missing data. All 312 retained questionnaires were complete, with no item-level missing values. The demographic characteristics are presented in Supplementary file 15.

Before CFA, the formal-survey data were screened for missingness, item distributions, and potential outliers. All 312 retained questionnaires were complete, and all item responses fell within the prespecified range of 1–5. Across the six POSPS items, skewness ranged from −0.871 to −0.214 and kurtosis from −1.233 to 0.792, indicating no severe univariate distributional departures. Floor effects were minimal (0.3–1.9%), although ceiling concentrations were observed across the six items (30.1–45.2%). A small number of item-level observations exceeded the criterion of |z| > 3.29; all represented valid responses within the response range and were therefore retained. Mahalanobis distance, evaluated at *p* < 0.001, identified two observations as potential multivariate outliers (D^2^ = 24.13 and 27.01). Mardia’s multivariate kurtosis indicated a slight departure from multivariate normality (−2.360, c.r. = − 2.128). Accordingly, CFA was estimated using MLR. A sensitivity analysis excluding the two observations flagged by Mahalanobis distance yielded substantively unchanged model fit, standardized factor loadings, and latent factor correlation; therefore, both observations were retained in the primary analysis. Detailed screening and sensitivity-analysis results are provided in Supplementary file 15.

**Figure 2:**
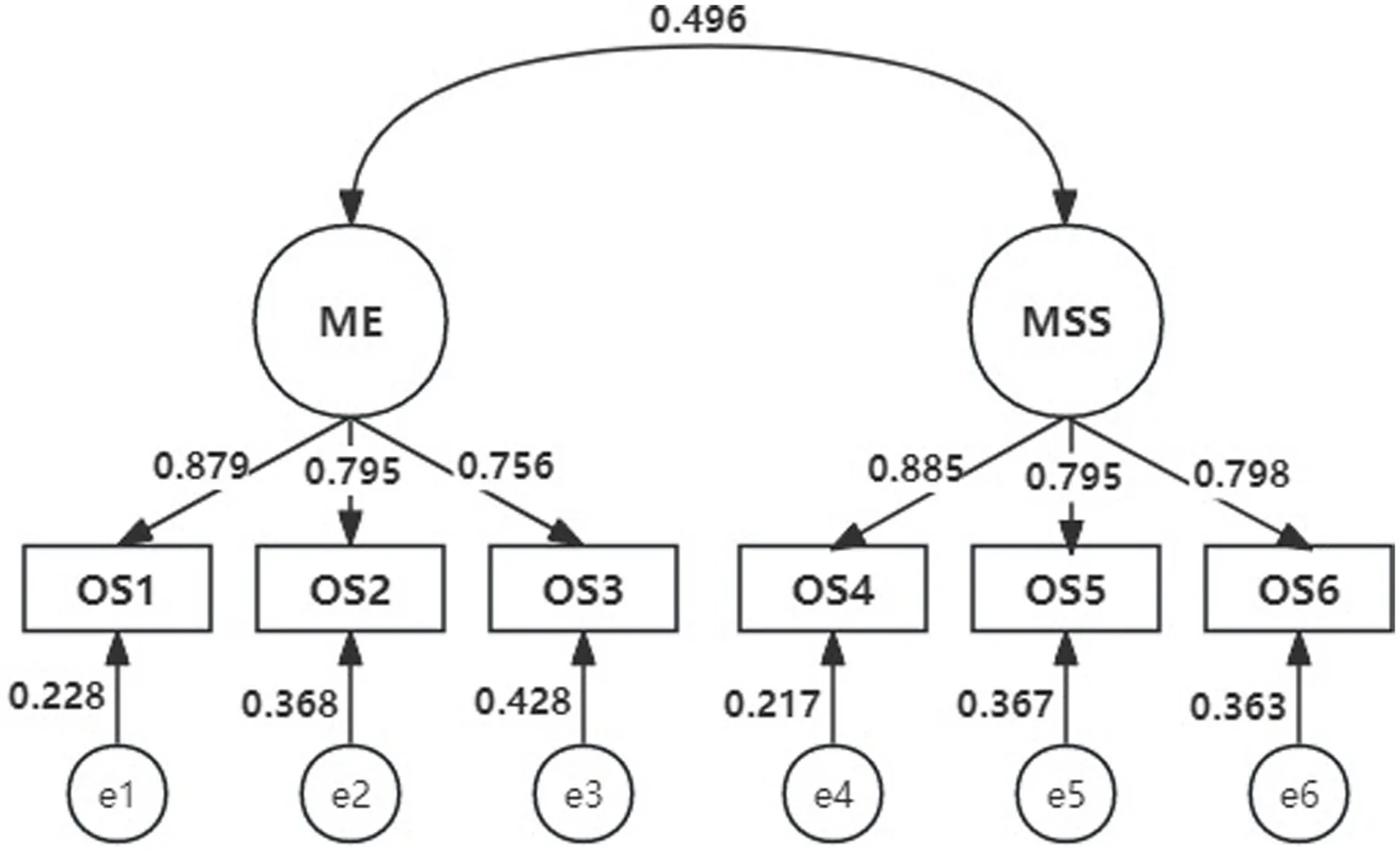
Xxx.

Two competing measurement models were specified to examine the factorial structure of the POSPS: a first-order single-factor model, in which all six items were specified as indicators of a single latent factor; and a correlated first-order two-factor model, in which OS1 - OS3 loaded on ME and OS4 - OS6 loaded on MSS. The correlated two-factor model showed substantially better fit than the competing single-factor model, χ^2^(8) = 19.808, χ^2^/df = 2.476, CFI = 0.989, TLI = 0.980, SRMR = 0.030, and RMSEA = 0.069 (Table 3). In addition, the two-factor model yielded substantially lower AIC (3993.656) and BIC (4064.773) values than the single-factor model (AIC = 4281.951; BIC = 4349.325). All six items loaded significantly on their intended factors, with standardized loadings ranging from 0.756 to 0.885 (all *p* < 0.001; Table 4). Residual variances ranged from 0.217 to 0.428, and item-level R^2^ values ranged from 0.572 to 0.783. The standardized latent correlation between ME and MSS was 0.496 (SE = 0.067, 95% CI [0.365, 0.626], *p* < 0.001), indicating that the two dimensions were moderately related but empirically distinguishable. The standardized correlated two-factor CFA model is shown in Figure 2. Accordingly, subsequent psychometric analyses and score interpretation were conducted at the subscale level; an overall six-item POSPS score was not recommended.

**Table 3:** Confirmatory factor analysis of the POSPS (*n* = 312).

Model	χ^2^	df	χ^2^/df	CFI	TLI	SRMR	RMSEA	AIC	BIC
First-order single-factor model	482.712	9	53.635	0.563	0.272	0.138	0.411	4281.951	4349.325
First-order two-factor model	19.808	8	2.476	0.989	0.980	0.030	0.069	3993.656	4064.773

**Table 4:** Standardized CFA parameter estimates and convergent validity of the POSPS (*N* = 312).

Construct	Item	Standardized factor loading	SE	95% CI	Residual variance	R^2^	CR	AVE	Square root of AVE
Medical Ethics Stigma Perception	OS1	0.879	0.025	[0.829, 0.928]	0.228	0.772	0.852	0.659	0.812
	OS2	0.795	0.031	[0.734, 0.856]	0.368	0.632			
	OS3	0.756	0.033	[0.691, 0.821]	0.428	0.572			
Medical Skills and Services Stigma Perception	OS4	0.885	0.023	[0.840, 0.930]	0.217	0.783	0.866	0.684	0.827
	OS5	0.795	0.026	[0.744, 0.847]	0.367	0.633			
	OS6	0.798	0.030	[0.740, 0.857]	0.363	0.637			

CFA was estimated using MLR. All standardized factor loadings were significant at *p* < 0.001. The standardized latent correlation between ME and MSS was 0.496 (SE = 0.067, 95% CI [0.365, 0.626], *p* < 0.001). CR, composite reliability; AVE, average variance extracted.

#### 4.4.2 Internal consistency

For descriptive completeness, the six-item item set yielded a Cronbach’s alpha of 0.841. However, because the CFA supported two related but distinct dimensions rather than a unidimensional structure, this coefficient was not interpreted as evidence supporting an overall POSPS score. Reliability was therefore evaluated at the subscale level. The three-item ME subscale demonstrated good internal consistency, with a Cronbach’s alpha of 0.849 and a McDonald’s omega of 0.851. The three-item MSS subscale showed similarly good internal consistency, with a Cronbach’s alpha of 0.862 and a McDonald’s omega of 0.862. These results supported acceptable internal consistency of both POSPS dimensions in the formal-survey sample.

#### 4.4.3 Validity test

Evidence for content validity was provided by the preceding qualitative interviews, two rounds of Delphi expert consultation, and target-population item pretesting. Structural validity was further supported by the correlated two-factor CFA in the formal survey. As shown in Table 4, standardized factor loadings ranged from 0.756 to 0.885, all exceeding the recommended threshold of 0.40. Convergent validity was assessed using CR and AVE. The CR values were 0.852 for ME and 0.866 for MSS, while the corresponding AVE values were 0.659 and 0.684, respectively. Both CR and AVE values exceeded commonly recommended criteria, supporting the convergent validity of the two dimensions. The square roots of the AVEs were 0.812 for ME and 0.827 for MSS. Both values exceeded the latent correlation between ME and MSS (0.496), satisfying the Fornell - Larcker criterion and supporting discriminant validity between the two dimensions. Nomological validity was examined using emotional exhaustion (20), ambivalent occupational identification (21), and job satisfaction (22) as theoretically relevant external constructs. ME was positively correlated with emotional exhaustion (r = 0.369, *p* < 0.001) and ambivalent occupational identification (r = 0.395, *p* < 0.001), and negatively correlated with job satisfaction (r = −0.422, *p* < 0.001). MSS showed the same pattern, with positive correlations with emotional exhaustion (r = 0.305, *p* < 0.001) and ambivalent occupational identification (r = 0.392, *p* < 0.001), and a negative correlation with job satisfaction (r = −0.454, *p* < 0.001). These associations were consistent with existing literature (33–35). The complete list of finalized scale items and their corresponding codes is presented in Table 5.

**Table 5:** The six-item Physician Occupational Stigma Perception Scale (POSPS).

Dimension	Item code	Item description
Medical Ethics Stigma Perception (ME)	OS1	I perceive that members of the public believe physicians profit from overtreatment, such as excessive examinations, overprescribing, or unnecessary surgery.
	OS2	I perceive that members of the public believe physicians provide attentive care only after receiving cash gifts (“red envelopes”) or other gifts from patients.
	OS3	I perceive that members of the public believe physicians give preferential care to patients with personal connections.
Medical Skills and Services Stigma Perception (MSS)	OS4	I perceive that patients and their families place excessive demands on physicians’ service attitudes while overlooking their professional expertise.
	OS5	I perceive that some patients and their families overlook the limitations of medicine and attribute treatment outcomes primarily to physicians’ clinical competence.
	OS6	I perceive that patients and their families interpret long waiting times and brief consultations as evidence that physicians are perfunctory or dismissive.

The POSPS was developed and psychometrically validated in Chinese. The English wording presented here is provided for reporting purposes and has not been independently validated as an English-language version. Items are rated on a five-point Likert scale from 1 (strongly disagree) to 5 (strongly agree).

## 5 Discussion

This study developed and psychometrically evaluated the Physician Occupational Stigma Perception Scale for use among Chinese physicians. Following an exploratory sequential mixed-methods design, the scale was generated from qualitative interviews, refined through two rounds of expert consultation and target-population pretesting, and subsequently examined in independent samples using exploratory and confirmatory factor analyses. The final POSPS consists of six items organized into two correlated dimensions: Medical Ethics Stigma Perception and Medical Skills and Services Stigma Perception. The two-factor structure was supported by both EFA and CFA, and both subscales demonstrated acceptable internal consistency, convergent validity, discriminant validity, and nomological validity. These findings provide preliminary evidence that the POSPS offers a concise and contextually grounded measure of physicians’ perceptions of occupational stigma in China.

A notable finding is that physicians’ perceived occupational stigma was represented by two distinct but related domains rather than by a single undifferentiated construct. The ME dimension captures perceived negative public judgments concerning physicians’ professional ethics, including suspicions of unnecessary treatment for financial gain, acceptance of informal benefits, and preferential treatment based on personal relationships. The MSS dimension reflects perceived devaluation of physicians’ professional competence and service, including excessive emphasis on service attitudes, attribution of treatment outcomes primarily to physicians’ clinical competence, and interpretations of brief consultations or long waiting times as evidence of perfunctory or dismissive care. This distinction is theoretically consistent with social-cognitive approaches to stigma, which conceptualize stigma as arising when socially salient labels become associated with negative stereotypes, evaluative judgments, and discriminatory responses (15, 17). In the present context, the two dimensions suggest that physicians may experience occupational stigma through at least two qualitatively different forms of public devaluation: questioning of their moral intentions and questioning of their professional competence and service behavior.

The content of the final POSPS also reflects features of the physician - public relationship in the Chinese healthcare context. The qualitative phase initially identified a broader range of stigma-related experiences, including seniority bias, gender-related prejudice, physical or disease-related stigma, occupational misunderstanding, and generalized stigma consciousness. However, not all of these experiences formed stable factors during quantitative refinement. Items reflecting generalized stigma consciousness and some socially contingent experiences showed weak communalities, unstable or conceptually inconsistent loadings, or insufficient physician specificity and were therefore removed during the iterative EFA process. The resulting six-item structure should consequently be understood as measuring the two most psychometrically coherent domains identified in the present samples rather than as representing every possible manifestation of stigma encountered by physicians. This distinction is important because occupational stigma can be heterogeneous and context dependent, and different forms of stigma may be more salient for particular specialties, career stages, genders, disease contexts, or healthcare settings.

The confirmatory analysis further supported the distinction between the two POSPS dimensions. The correlated two-factor model fit the formal-survey data substantially better than the competing single-factor model, and the latent correlation between ME and MSS was moderate rather than excessively high. Convergent validity was supported by substantial standardized factor loadings and acceptable composite reliability and average variance extracted for both dimensions. At the same time, the square root of the AVE for each dimension exceeded the correlation between the two latent factors, supporting their empirical distinctiveness. These findings suggest that perceptions of ethical stigma and perceptions of skills-and-services stigma share a common occupational context but capture nonredundant aspects of physicians’ stigma experiences. Accordingly, the POSPS is best interpreted at the subscale level. Although the six items showed acceptable overall internal consistency descriptively, the markedly superior fit of the correlated two-factor model does not by itself justify treating all six items as a unidimensional score. We therefore do not recommend an overall POSPS score at the present stage.

Nomological validity was also supported by the associations between the two POSPS dimensions and theoretically relevant occupational outcomes. Both ME and MSS were positively associated with emotional exhaustion and ambivalent occupational identification and negatively associated with job satisfaction. These patterns are consistent with previous research showing that perceived occupational stigma may be accompanied by greater emotional exhaustion and more conflicted or ambivalent forms of occupational identification, while also being associated with less favorable occupational attitudes. Prior studies have reported positive associations between occupational stigma perception and emotional exhaustion (33) and between occupational stigma perception and ambivalent occupational identification (35). More broadly, research on stigmatized occupations suggests that employees may simultaneously experience attachment to their occupation and discomfort with the negative social meanings attached to it, resulting in complex rather than uniformly negative identity responses. Thus, the present pattern of correlations supports the theoretical relevance of the POSPS to physicians’ occupational well-being while also indicating that stigma perception is related to emotional and identity-related outcomes.

The POSPS has several potential research and practical applications. First, its brevity may facilitate inclusion in physician surveys in which occupational stigma is examined alongside burnout, professional identity, job satisfaction, or other occupational-health indicators. Second, separating ME from MSS may help distinguish different sources of perceived public devaluation that would be obscured by a single global stigma score. For example, interventions aimed at mistrust surrounding physicians’ financial or ethical motives may require different organizational and communication strategies from those addressing unrealistic expectations regarding treatment outcomes, consultation time, or service behavior. Third, because the items explicitly assess physicians’ perceptions of negative public evaluations, rather than physicians’ own endorsement of those evaluations, the scale may be useful for examining how perceived social stigma is associated with physicians’ occupational experiences.

Several limitations should be acknowledged. First, although participants were recruited from geographically diverse settings, the quantitative samples were obtained primarily from tertiary public hospitals using purposive and referral-assisted recruitment. The samples therefore cannot be considered nationally representative of Chinese physicians. Future studies should examine the POSPS among physicians working in primary healthcare institutions, secondary hospitals, private hospitals, rural settings, and different regions of China. Second, the final instrument contains only three items per dimension. The short format improves feasibility but inevitably narrows content coverage. Experiences related to seniority, gender, infectious-disease stigma, specialty-specific stereotypes, and broader stigma consciousness were not represented in the final six-item scale. Future studies may determine whether these experiences represent context-specific extensions of the POSPS or distinct forms of physician occupational stigma. Third, the psychometric evidence was based primarily on cross-sectional self-report data. The observed associations with emotional exhaustion, ambivalent occupational identification, and job satisfaction therefore should not be interpreted causally. Longitudinal studies are needed to examine temporal relationships between perceived occupational stigma and physician well-being and occupational attitudes. Test - retest reliability was also not examined, and future research should assess temporal stability and responsiveness to changes in occupational or organizational conditions. Fourth, although the two-factor structure was replicated in an independent formal-survey sample, further validation in independent populations is warranted, including examination of measurement equivalence across relevant subgroups such as gender, career stage, specialty, hospital level, and geographic region. Finally, the POSPS was developed specifically in the Chinese sociocultural and healthcare context. Cross-cultural adaptation and validation would be necessary before applying the scale to physicians in other countries or healthcare systems.

Despite these limitations, the present study provides a systematic initial measure of perceived occupational stigma among Chinese physicians. By integrating physicians’ qualitative accounts with expert evaluation and psychometric testing, the POSPS identifies two related but distinguishable domains of perceived occupational stigma concerning medical ethics and medical skills and services. The six-item scale demonstrated acceptable reliability and multiple forms of validity in the present samples and may provide a useful foundation for future research on occupational stigma and physicians’ occupational well-being in China.

## 6 Conclusion

This study developed and psychometrically evaluated the six-item Physician Occupational Stigma Perception Scale, which was developed and administered in Chinese among physicians in China. The scale comprises two related but distinct dimensions, Medical Ethics Stigma Perception and Medical Skills and Services Stigma Perception. The findings provide preliminary support for the reliability and construct validity of the two POSPS subscales in the present samples. The two subscales should be interpreted separately rather than combined into an overall POSPS score. The POSPS provides a brief, contextually grounded measure for examining physicians’ perceptions of occupational stigma and their associations with occupational well-being in China.

## Data Availability

The original contributions presented in the study are included in the article/Supplementary material, further inquiries can be directed to the corresponding author.
