# Supplementary material for: Development and psychometric validation of the Physician Occupational Stigma Perception Scale among Chinese physicians
Source: Front Public Health. 2026 Sep 11;14:1912387. doi: 10.3389/fpubh.2026.1912387 (PMC13612318; doi:10.3389/fpubh.2026.1912387)
Supplement: Supplementary file 1 [file Table_1.DOCX]

## Supplementary File 1 Interview Guide for the Qualitative Phase

### Introduction

Before the interview, the researcher briefly introduced the purpose of the study, explained the interview procedure, and obtained informed consent. Participants were informed that the interview focused on their perceptions and experiences of occupational stigma toward physicians and that their responses would be used solely for academic research.

### Core Interview Questions

**1. General perception of the medical profession**

Do you consider being a physician a good or a bad occupation? What do you think are the positive and negative aspects of this profession?

**2. Perceived occupational stigma toward physicians**

Compared with other occupations, what kinds of stigma, prejudice, discrimination, stereotypes, or other negative labels do physicians encounter? Please provide specific examples.

**3. Perceptions and interpretations of stigmatizing experiences**

When you encounter stereotypes, prejudice, misunderstanding, discrimination, or other forms of occupational stigma toward physicians, what are your thoughts about these experiences?

### Interviewer Notes

The interviews were semistructured and primarily based on open-ended questions. When necessary, follow-up probes were used to encourage participants to clarify their responses or provide specific examples. The interviewer avoided suggesting predefined categories or responses so that participants could describe occupational stigma in their own words.
