# Supplementary material for: Development and psychometric validation of the Physician Occupational Stigma Perception Scale among Chinese physicians
Source: Front Public Health. 2026 Sep 11;14:1912387. doi: 10.3389/fpubh.2026.1912387 (PMC13612318; doi:10.3389/fpubh.2026.1912387)
Supplement: Supplementary file 2 [file Table_10.DOCX]

**Supplementary file 10 Sensitivity analysis of the EFA results after exclusion of the potential multivariate outlier.**

| **Indicator** | **Full sample (N=193)** | **Case 165 excluded (N=192)** |
| --- | --- | --- |
| Initial 12-item Horn parallel analysis | 3 factors | 3 factors |
| Initial KMO | 0.803 | 0.807 |
| Core Factor 1 | OS1–OS3 | OS1–OS3 |
| Core Factor 2 | OS4–OS6 | OS4–OS6 |
| OS12 communality | 0.030 | 0.027 |
| Initial F1–F2 correlation | 0.498 | 0.502 |
| Final 6-item Horn parallel analysis | 2 factors | 2 factors |
| Final KMO | 0.780 | 0.781 |
| OS1 loading | 0.889 | 0.885 |
| OS2 loading | 0.738 | 0.745 |
| OS3 loading | 0.811 | 0.813 |
| OS4 loading | 0.904 | 0.884 |
| OS5 loading | 0.784 | 0.810 |
| OS6 loading | 0.733 | 0.710 |
| Final F1–F2 correlation | 0.470 | 0.477 |

**Note:** The sensitivity analysis excluded the single participant whose Mahalanobis distance exceeded the χ²(12) criterion at p < 0.001. Factor retention was determined using Horn’s parallel analysis. EFA used principal axis factoring with Promax rotation.
