# Supplementary material for: Development and psychometric validation of the Physician Occupational Stigma Perception Scale among Chinese physicians
Source: Front Public Health. 2026 Sep 11;14:1912387. doi: 10.3389/fpubh.2026.1912387 (PMC13612318; doi:10.3389/fpubh.2026.1912387)
Supplement: Supplementary file 3 [file Table_11.DOCX]

**Supplementary file 11 Parallel analysis across the iterative item-refinement sequence.**

| **Item set** | **Items retained** | **Observed eigenvalues used for the retention decision** | **95th-percentile random eigenvalues** | **Factors retained** |
| --- | --- | --- | --- | --- |
| Initial | 12 | 4.031, 1.508, 1.373, 1.106 | 1.545, 1.391, 1.290, 1.201 | 3 |
| After OS12 removal | 11 | 3.997, 1.504, 1.373, 1.105 | 1.516, 1.356, 1.259, 1.172 | 3 |
| After OS7 removal | 10 | 3.920, 1.497, 1.262, 0.874 | 1.483, 1.325, 1.225, 1.146 | 3 |
| After OS8 removal | 9 | 3.642, 1.479, 1.255, 0.749 | 1.451, 1.302, 1.199, 1.109 | 3 |
| After OS11 removal | 8 | 3.618, 1.389, 1.007, 0.649 | 1.409, 1.268, 1.162, 1.078 | 2 |
| After OS10 removal | 7 | 3.554, 1.377, 0.725 | 1.374, 1.227, 1.124 | 2 |
| Final, after OS9 removal | 6 | 3.280, 1.372, 0.468 | 1.343, 1.196, 1.088 | 2 |

**Note:** A factor was retained when the observed eigenvalue exceeded the corresponding 95th-percentile eigenvalue from the random datasets. Only the eigenvalues needed to identify the point at which retention ceased are displayed.
