# Supplementary material for: Development and psychometric validation of the Physician Occupational Stigma Perception Scale among Chinese physicians
Source: Front Public Health. 2026 Sep 11;14:1912387. doi: 10.3389/fpubh.2026.1912387 (PMC13612318; doi:10.3389/fpubh.2026.1912387)
Supplement: Supplementary file 4 [file Table_12.DOCX]

**Supplementary file 12 Initial three-factor EFA of the 12-item preliminary POSPS (N=193).**

| **Item** | **Factor 1** | **Factor 2** | **Factor 3** | **Communality (h²)** |
| --- | --- | --- | --- | --- |
| OS1 | 0.860 | — | — | 0.743 |
| OS2 | 0.754 | — | — | 0.610 |
| OS3 | 0.840 | — | — | 0.651 |
| OS4 | — | 0.877 | — | 0.755 |
| OS5 | — | 0.796 | — | 0.611 |
| OS6 | — | 0.685 | — | 0.584 |
| OS7 | — | — | 0.351 | 0.192 |
| OS8 | 0.327 | 0.306 | — | 0.310 |
| OS9 | 0.358 | 0.333 | −0.347 | 0.425 |
| OS10 | — | — | 0.579 | 0.380 |
| OS11 | — | — | 0.378 | 0.159 |
| OS12 | — | — | — | 0.030 |
