# Supplementary material for: Development and psychometric validation of the Physician Occupational Stigma Perception Scale among Chinese physicians
Source: Front Public Health. 2026 Sep 11;14:1912387. doi: 10.3389/fpubh.2026.1912387 (PMC13612318; doi:10.3389/fpubh.2026.1912387)
Supplement: Supplementary file 5 [file Table_13.DOCX]

**Supplementary file 13 Iterative EFA and item-refinement process for the preliminary POSPS.**

| **Step** | **Item set** | **KMO** | **Bartlett’s test** | **Factors retained by PA** | **Item removed** | **Communality (h²)** | **Primary loading** | **Basis for removal** |
| --- | --- | --- | --- | --- | --- | --- | --- | --- |
| Initial EFA | 12 items | 0.803 | χ²(66)=772.262, p<0.001 | 3 | **OS12** | **0.030** | <\|0.30\| | Extremely low communality and no salient loading |
| Iteration 1 | 11 items | 0.804 | χ²(55)=762.279, p<0.001 | 3 | **OS7** | **0.164** | **0.309** | Low communality and weak primary loading |
| Iteration 2 | 10 items | 0.805 | χ²(45)=719.961, p<0.001 | 3 | **OS8** | **0.264** | **0.354** | Low communality, weak loading, and conceptually incongruent factor assignment |
| Iteration 3 | 9 items | 0.790 | χ²(36)=658.000, p<0.001 | 3 | **OS11** | **0.170** | **0.400** | Very low communality despite a marginal primary loading |
| Iteration 4 | 8 items | 0.807 | χ²(28)=621.577, p<0.001 | **2** | **OS10** | **0.057** | <\|0.30\| | After PA supported two factors, the item showed extremely low communality and no salient loading on either retained factor |
| Iteration 5 | 7 items | 0.808 | χ²(21)=604.229, p<0.001 | 2 | **OS9** | **0.241** | **0.349** | Low communality, weak loading, and conceptual inconsistency with the medical ethics factor |
| Final EFA | ****6 items**** | 0.780 | χ²(15)=557.609, p<0.001 | **2** | — | **0.588–0.793** | **0.733–0.904** | All six items showed strong primary loadings, adequate communalities, and no salient cross-loadings |

****N**ote:** EFA was conducted using principal axis factoring with Promax rotation. Pattern coefficients with absolute values below .30 were suppressed. Factor retention was determined primarily by Horn’s parallel analysis (PA), together with inspection of the scree plot and theoretical interpretability. Items were removed one at a time, and the factor-retention decision and EFA solution were re-evaluated after each removal. Item-removal decisions considered communalities, the magnitude and specificity of primary loadings, cross-loadings, and conceptual coherence. POSPS, Physician Occupational Stigma Perception Scale.
