# Supplementary material for: Development and psychometric validation of the Physician Occupational Stigma Perception Scale among Chinese physicians
Source: Front Public Health. 2026 Sep 11;14:1912387. doi: 10.3389/fpubh.2026.1912387 (PMC13612318; doi:10.3389/fpubh.2026.1912387)
Supplement: Supplementary file 6 [file Table_14.DOCX]

**Supplementary file 14 EFA results before and after removal of OS8.**

**Panel A. Ten-item solution before removal of OS8**

| **Item** | **Factor 1** | **Factor 2** | **Factor 3** | **Communality (h²)** |
| --- | --- | --- | --- | --- |
| OS1 | 0.880 | — | — | 0.760 |
| OS2 | 0.735 | — | — | 0.579 |
| OS3 | 0.840 | — | — | 0.643 |
| OS4 | — | 0.896 | — | 0.780 |
| OS5 | — | 0.788 | — | 0.610 |
| OS6 | — | 0.697 | — | 0.583 |
| OS8 | 0.354 | — | — | 0.264 |
| OS9 | 0.394 | — | — | 0.309 |
| OS10 | — | — | 0.888 | 0.803 |
| OS11 | — | — | 0.440 | 0.202 |

**Note:** KMO = 0.805; Bartlett’s χ²(45) = 719.961, p < 0.001. Parallel analysis supported three factors.

**Panel B. Nine-item solution after removal of OS8**

| **Item** | **Factor 1** | **Factor 2** | **Factor 3** | **Communality (h²)** |
| --- | --- | --- | --- | --- |
| OS1 | .880 | — | — | .771 |
| OS2 | .742 | — | — | .598 |
| OS3 | .834 | — | — | .645 |
| OS4 | — | .897 | — | .781 |
| OS5 | — | .787 | — | .606 |
| OS6 | — | .715 | — | .604 |
| OS9 | .359 | — | — | .260 |
| OS10 | — | — | .991 | .987 |
| OS11 | — | — | .400 | .170 |

**Note:** KMO = 0.790; Bartlett’s χ²(36) = 658.000, p < 0.001. Parallel analysis continued to support three factors. Principal axis factoring with Promax rotation was used in both panels; pattern coefficients with absolute values below 0.30 were suppressed.
