# Supplementary material for: Development and psychometric validation of the Physician Occupational Stigma Perception Scale among Chinese physicians
Source: Front Public Health. 2026 Sep 11;14:1912387. doi: 10.3389/fpubh.2026.1912387 (PMC13612318; doi:10.3389/fpubh.2026.1912387)
Supplement: Supplementary file 7 [file Table_15.DOCX]

**Supplementary file 15a Demographic characteristics of participants in the formal survey (N = 312).**

| **Variable** | **Category** | **n** | **%** |
| --- | --- | --- | --- |
| Gender | Female | 150 | 48.08% |
|  | Male | 162 | 51.92% |
| Age group | ≤30 years | 126 | 40.38% |
|  | 31–40 years | 142 | 45.51% |
|  | 41–50 years | 33 | 10.58% |
|  | >50 years | 11 | 3.53% |
| Education | Bachelor’s degree or below | 106 | 33.97% |
|  | Master’s degree | 159 | 50.96% |
|  | Doctoral degree | 47 | 15.06% |
| Years of professional experience | ≤5 years | 115 | 36.86% |
|  | 6–10 years | 104 | 33.33% |
|  | 11–15 years | 79 | 25.32% |
|  | 16–20 years | 14 | 4.49% |
| Professional title | None | 48 | 15.38% |
|  | Junior | 88 | 28.21% |
|  | Intermediate | 110 | 35.26% |
|  | Senior | 66 | 21.15% |
| Total |  | 312 | 100.00 |

**Supplementary file 15b Distributional characteristics of the POSPS items in the formal psychometric-validation sample (N = 312).**

| **Item** | **Mean** | **SD** | **Min** | **Max** | **Skewness** | **Kurtosis** | **Floor (%)** | **Ceiling (%)** |
| --- | --- | --- | --- | --- | --- | --- | --- | --- |
| OS1 | 3.96 | 0.906 | 1 | 5 | -0.810 | 0.744 | 1.9 | 30.1 |
| OS2 | 4.06 | 0.937 | 1 | 5 | -0.871 | 0.578 | 1.9 | 38.5 |
| OS3 | 3.95 | 0.934 | 1 | 5 | -0.708 | 0.347 | 1.9 | 32.1 |
| OS4 | 4.05 | 0.802 | 1 | 5 | -0.688 | 0.792 | 1.0 | 30.4 |
| OS5 | 4.03 | 0.893 | 1 | 5 | -0.214 | −1.233 | 0.3 | 39.4 |
| OS6 | 4.17 | 0.897 | 1 | 5 | -0.853 | 0.362 | 1.3 | 45.2 |

**Note:** No item-level missing data were observed. Floor and ceiling effects represent the percentages of respondents selecting the lowest and highest response categories, respectively. A small number of item-level observations exceeded |z| > 3.29, but all represented valid responses within the 1-5 range. Mardia’s multivariate kurtosis was -2.360 (c.r. = -2.128). Two observations exceeded the Mahalanobis-distance criterion at p < 0.001; the corresponding sensitivity analysis is reported in Supplementary file 15c.

**Supplementary file 15c Sensitivity analysis of the CFA after exclusion of the two potential multivariate outliers.**

| **Parameter** | **Full sample (N=312)** | **Sensitivity sample (N=310)** |
| --- | --- | --- |
| χ² | 19.808 | 20.526 |
| df | 8 | 8 |
| CFI | 0.989 | 0.989 |
| TLI | 0.980 | 0.979 |
| RMSEA | 0.069 | 0.071 |
| SRMR | 0.030 | 0.032 |
| OS1 loading | 0.879 | 0.881 |
| OS2 loading | 0.795 | 0.790 |
| OS3 loading | 0.756 | 0.749 |
| OS4 loading | 0.885 | 0.879 |
| OS5 loading | 0.795 | 0.811 |
| OS6 loading | 0.798 | 0.781 |
| ME–MSS latent correlation | 0.496 | 0.482 |

**Note:** Two observations were flagged as potential multivariate outliers using Mahalanobis distance at p < 0.001 (D² = 24.13 and 27.01). Excluding these observations did not materially alter global model fit, standardized factor loadings, or the latent correlation between ME and MSS. The full N=312 sample was therefore retained for the primary analyses. AIC and BIC were not compared across the two analyses because they were based on different samples.
