# Supplementary material for: Development and psychometric validation of the Physician Occupational Stigma Perception Scale among Chinese physicians
Source: Front Public Health. 2026 Sep 11;14:1912387. doi: 10.3389/fpubh.2026.1912387 (PMC13612318; doi:10.3389/fpubh.2026.1912387)
Supplement: Supplementary file 8 [file Table_2.DOCX]

**Supplementary file 2 Demographic Information of Interviewees (n=23)**

| **Variable** | **Category** | **n** | **%** |
| --- | --- | --- | --- |
| **Gender** | Male | 12 | 52.2 |
|  | Female | 11 | 47.8 |
| **Education level** | Doctoral degree | 10 | 43.5 |
|  | Master’s degree | 13 | 56.5 |
| **Age** | ≤30 | 7 | 30.4 |
|  | 31–40 | 9 | 39.1 |
|  | 41–50 | 6 | 26.1 |
|  | >50 | 1 | 4.3 |
| **Professional rank** | Chief physician | 6 | 26.1 |
|  | Deputy chief physician | 6 | 26.1 |
|  | Attending physician | 9 | 39.1 |
|  | Resident physician | 2 | 8.7 |
| Total |  | 23 | 100 |
