# Supplementary material for: Development and psychometric validation of the Physician Occupational Stigma Perception Scale among Chinese physicians
Source: Front Public Health. 2026 Sep 11;14:1912387. doi: 10.3389/fpubh.2026.1912387 (PMC13612318; doi:10.3389/fpubh.2026.1912387)
Supplement: Supplementary file 9 [file Table_3.DOCX]

**Supplementary file 3 Coding process**

| **Open coding** | **Axial coding** | **Selective coding** |
| --- | --- | --- |
| a1 Distrust of younger physicians; a2 Preference for senior physicians; a3 Preference for chief physicians | **A1 Seniority bias** | **Social stigma perception** |
| a4 Preference for female physicians; a5 Preference for male physicians | **A2 Gender discrimination** |  |
| a6 Fear of infection transmission or contact with patients with stigmatized diseases | **A3 Physical stigma perception** |  |
| a7 Excessive examinations; a8 Use of expensive medications; a9 Overprescribing; a10 Exaggeration of illness severity; a11 Unnecessary surgery | **A4 Overtreatment** | **Moral stigma perception** |
| a12 Expectation of giving physicians red envelopes or gifts; a13 Unreasonable medical charges; a14 Kickbacks | **A5 Improper financial gain** |  |
| a15 Seeking care through acquaintances; a16 Reliance on personal connections | **A6 Guanxi-based care** |  |
| a17 Treating physicians as service staff; a18 Poor service attitude; a19 Neglect of professional expertise | **A7 Service expectations that neglect professional expertise** | **Medical skills and services stigma perception** |
| a20 Excessively high treatment expectations; a21 Perceived inadequate physician competence; a22 Knowledge asymmetry | **A8 Attribution of treatment outcomes to physicians’ skills** |  |
| a23 Long waiting times; a24 Short consultation times; a25 Perceived perfunctory care | **A9 Perceived perfunctoriness** |  |
| a26 Belief that physicians are wealthy; a27 Belief that physicians receive free medical care | **A10 Misunderstanding of the occupation** | **Misunderstanding** |
| a28 Expectation of complete recovery after surgery; a29 Expectation of immediate recovery after medication | **A11 Misunderstanding of treatment effects** |  |

**Note:** In the specific content of the dimension of perception of misunderstandings, the misunderstanding of the occupation refers to the positive label evaluation of doctors. The misunderstanding of treatment effects is the cognitive error of patients regarding the efficacy of drugs and surgeries. This does not conform to the connotation and extension of occupational stigma perception, and therefore is not included in the item pool.
