# Supplementary material for: Development and psychometric validation of the Physician Occupational Stigma Perception Scale among Chinese physicians
Source: Front Public Health. 2026 Sep 11;14:1912387. doi: 10.3389/fpubh.2026.1912387 (PMC13612318; doi:10.3389/fpubh.2026.1912387)
Supplement: Supplementary file 10 [file Table_4.DOCX]

**Supplementary file 4 Initial 15-item candidate pool of the POSPS before Delphi expert consultation.**

| **Provisional content domain** | **Candidate code** | **Item Description** | **Item source** |
| --- | --- | --- | --- |
| **Moral stigma perception** | **C1** | **Physicians are often perceived as profiting from overtreatment, such as excessive examinations, overprescribing, or unnecessary surgery.** | Interview |
|  | **C2** | Physicians are often perceived as providing attentive care only after receiving cash gifts (“red envelopes”) or other gifts. |  |
|  | **C3** | Physicians are often perceived as giving preferential care to patients with personal connections. |  |
| **Medical skills and services stigma perception** | **C4** | Physicians are often subject to excessive demands regarding service attitude, while their professional expertise is overlooked. |  |
|  | **C5** | Some patients and their families fail to recognize the limitations of medicine and attribute treatment outcomes directly to physicians’ medical skills. |  |
|  | **C6** | Because of long waiting times and brief consultations, physicians are often perceived as perfunctory or dismissive toward patients. |  |
| **Social stigma perception** | **C7** | **People believe that older physicians are more professional.** |  |
|  | **C8** | **People believe that physicians of a particular gender are more reliable in certain specialties (e.g., female physicians are considered more professional in obstetrics and gynecology).** |  |
|  | **C9** | Physicians are negatively viewed because of the risk of infection associated with occupational exposure or contact with patients who are themselves stigmatized. |  |
| **Generalized stigma consciousness** | **C10** | Many people outside our profession hold negative views of our profession, even if they do not express them openly. | Adapted from the Stigma Consciousness Questionnaire (SCQ) |
|  | **C11** | Many people outside our profession judge us on the basis of our work and job responsibilities. |  |
|  | **C12** | **Many people outside our profession find it difficult to view us equally.** |  |
|  | **C13** | **My occupation affects how others perceive me.** |  |
|  | **C14** | **When communicating with people outside our profession, I feel that they view and interpret my behavior on the basis of my occupation.** |  |
|  | **C15** | Public stereotypes about my occupation have a substantial impact on me personally. |  |
