# Supplementary material for: Development and psychometric validation of the Physician Occupational Stigma Perception Scale among Chinese physicians
Source: Front Public Health. 2026 Sep 11;14:1912387. doi: 10.3389/fpubh.2026.1912387 (PMC13612318; doi:10.3389/fpubh.2026.1912387)
Supplement: Supplementary file 11 [file Table_5.DOCX]

**Supplementary file 5 Basic Information of Experts in the Delphi Consultation (N = 13)**

| **Variable** | **Category** | **n** | **%** |
| --- | --- | --- | --- |
| Sex | Male | 7 | 53.85 |
|  | Female | 6 | 46.15 |
| Age | 31–40 | 3 | 23.08 |
|  | 41–50 | 5 | 38.46 |
|  | 51–60 | 5 | 38.46 |
| Years of Work | 6–10 | 3 | 23.08 |
|  | 11–20 | 5 | 38.46 |
|  | 21–30 | 3 | 23.08 |
|  | Over 30 | 2 | 15.38 |
| Professional Title | Senior Title | 9 | 69.23 |
|  | Associate Senior Title | 4 | 30.77 |
| Education Level | Master’s Degree | 3 | 23.08 |
|  | Doctoral Degree | 10 | 76.92 |
| Field of Expertise | Management | 3 | 23.08 |
|  | Medical Specialty | 10 | 76.92 |
| **Total** |  | **13** | **100.00** |
