# Supplementary material for: Development and psychometric validation of the Physician Occupational Stigma Perception Scale among Chinese physicians
Source: Front Public Health. 2026 Sep 11;14:1912387. doi: 10.3389/fpubh.2026.1912387 (PMC13612318; doi:10.3389/fpubh.2026.1912387)
Supplement: Supplementary file 12 [file Table_6.DOCX]

**Supplementary file 6 Item scores from the first round of Delphi expert consultation.**

| **Dimension** | **Code** | **Item Description** | **Mean** | **SD** | **CV** | **Full Score Rate (%)** |
| --- | --- | --- | --- | --- | --- | --- |
| **Moral stigma perception** | **C1** | **Physicians are often perceived as profiting from overtreatment, such as excessive examinations, overprescribing, or unnecessary surgery.** | 4.92 | 0.277 | 0.06 | 92.31 |
|  | **C2** | Physicians are often perceived as providing attentive care only after receiving cash gifts (“red envelopes”) or other gifts. | 5.00 | 0.000 | 0.00 | 100.00 |
|  | **C3** | Physicians are often perceived as giving preferential care to patients with personal connections. | 4.92 | 0.277 | 0.06 | 92.31 |
| **Medical skills and services stigma perception** | **C4** | Physicians are often subject to excessive demands regarding service attitude, while their professional expertise is overlooked. | 4.92 | 0.277 | 0.06 | 92.31 |
|  | **C5** | Some patients and their families fail to recognize the limitations of medicine and attribute treatment outcomes directly to physicians’ medical skills. | 5.00 | 0.000 | 0.00 | 100.00 |
|  | **C6** | Because of long waiting times and brief consultations, physicians are often perceived as perfunctory or dismissive toward patients. | 4.92 | 0.277 | 0.06 | 92.31 |
| **Social stigma perception** | **C7** | **People believe that older physicians are more professional.** | 3.92 | 0.494 | 0.13 | 7.69 |
|  | **C8** | **People believe that physicians of a particular gender are more reliable in certain specialties (e.g., female physicians are considered more professional in obstetrics and gynecology).** | 3.92 | 0.494 | 0.13 | 7.69 |
|  | **C9** | Physicians are negatively viewed because of the risk of infection associated with occupational exposure or contact with patients who are themselves stigmatized. | 3.92 | 0.494 | 0.13 | 7.69 |
| **Generalized stigma consciousness (SCQ-derived)** | **C10** | Many people outside our profession hold negative views of our profession, even if they do not express them openly. | 4.00 | 0.408 | 0.10 | 7.69 |
|  | **C11** | Many people outside our profession judge us on the basis of our work and job responsibilities. | 2.46 | 0.519 | 0.21 | 0.00 |
|  | **C12** | **Many people outside our profession find it difficult to view us equally.** | 3.85 | 0.555 | 0.14 | 7.69 |
|  | **C13** | **My occupation affects how others perceive me.** | 2.85 | 0.376 | 0.13 | 0.00 |
|  | **C14** | **When communicating with people outside our profession, I feel that they view and interpret my behavior on the basis of my occupation.** | 2.85 | 0.376 | 0.13 | 0.00 |
|  | **C15** | Public stereotypes about my occupation have a substantial impact on me personally. | 3.92 | 0.641 | 0.16 | 15.38 |

Note: The item wording presented in this table reflects the 15-item candidate pool evaluated in the first Delphi round, before subsequent wording refinement. “Moral stigma perception” was the provisional domain label used at this stage and was subsequently renamed “Medical ethics stigma perception.” The POSPS was developed in Chinese; the English wording shown here is a faithful rendering of the Chinese candidate items. CV = coefficient of variation.
