# Supplementary material for: Development and psychometric validation of the Physician Occupational Stigma Perception Scale among Chinese physicians
Source: Front Public Health. 2026 Sep 11;14:1912387. doi: 10.3389/fpubh.2026.1912387 (PMC13612318; doi:10.3389/fpubh.2026.1912387)
Supplement: Supplementary file 13 [file Table_7.DOCX]

**Supplementary file 7 Item scores from the second round of Delphi expert consultation.**

| **Dimension** | **Original code** | **Current code** | **Item Description** | **Mean** | **SD** | **CV** | **Full Score Rate (%)** |
| --- | --- | --- | --- | --- | --- | --- | --- |
| ****Medical ethics stigma perception**** | C1 | OS1 | I perceive that members of the public believe physicians profit from overtreatment, such as excessive examinations, overprescribing, or unnecessary surgery. | 5.00 | 0.00 | 0.00 | 100.00 |
|  | C2 | OS2 | I perceive that members of the public believe physicians provide attentive care only after receiving cash gifts (“red envelopes”) or other gifts from patients. | 5.00 | 0.00 | 0.00 | 100.00 |
|  | C3 | OS3 | I perceive that members of the public believe physicians give preferential care to patients with personal connections. | 5.00 | 0.00 | 0.00 | 100.00 |
| ****Medical skills and services stigma perception**** | C4 | OS4 | I perceive that patients and their families place excessive demands on physicians’ service attitudes while overlooking their professional expertise. | 4.90 | 0.32 | 0.06 | 90.00 |
|  | C5 | OS5 | I perceive that some patients and their families overlook the limitations of medicine and attribute treatment outcomes primarily to physicians’ clinical competence. | 5.00 | 0.00 | 0.00 | 100.00 |
|  | C6 | OS6 | I perceive that patients and their families interpret long waiting times and brief consultations as evidence that physicians are perfunctory or dismissive. | 4.90 | 0.32 | 0.06 | 90.00 |
| ****Social stigma perception**** | C7 | OS7 | I perceive that younger or less experienced physicians are sometimes subject to prejudice because of their age or limited seniority. | 4.00 | 0.47 | 0.12 | 10.00 |
|  | C8 | OS8 | I perceive that physicians may experience gender-based discrimination, such as patients and their families preferring female physicians in obstetrics and gynecology. | 4.00 | 0.47 | 0.12 | 10.00 |
|  | C9 | OS9 | I perceive that physicians may be viewed negatively because of occupational exposure to infection or contact with patients who are themselves stigmatized. | 3.90 | 0.32 | 0.08 | 0.00 |
| ****Generalized stigma consciousness (SCQ-derived)**** | C10 | OS10 | I perceive that many people outside the medical profession hold negative views of physicians, even when they do not express them openly. | 4.00 | 0.47 | 0.12 | 10.00 |
|  | C12 | OS11 | I perceive that many people outside the medical profession find it difficult to view physicians objectively. | 3.80 | 0.63 | 0.17 | 10.00 |
|  | C15 | OS12 | I perceive that public stereotypes about the medical profession have a strong personal impact on me. | 4.20 | 0.42 | 0.10 | 20.00 |

**Note:** The Chinese questionnaire at this stage used an introductory perceptual frame equivalent to “In my work, I perceive/experience…”. For clarity in the English presentation, this perceptual frame is incorporated into each item below. This wording clarification does not represent a post hoc change to the Chinese items rated by the experts. CV = coefficient of variation. SCQ = Stigma Consciousness Questionnaire.
