# Supplementary material for: Development and psychometric validation of the Physician Occupational Stigma Perception Scale among Chinese physicians
Source: Front Public Health. 2026 Sep 11;14:1912387. doi: 10.3389/fpubh.2026.1912387 (PMC13612318; doi:10.3389/fpubh.2026.1912387)
Supplement: Supplementary file 14 [file Table_8.DOCX]

**Supplementary file 8 Demographic characteristics of physicians in the scale-refinement sample (n=193)**

| **Variable** | **Category** | **n** | **%** |
| --- | --- | --- | --- |
| Gender | Male | 99 | 51.30 |
|  | Female | 94 | 48.70 |
| Age | ≤30 | 96 | 49.74 |
|  | 31–40 | 68 | 35.23 |
|  | 41–50 | 23 | 11.92 |
|  | >50 | 6 | 3.11 |
| Years of Work | ≤5 | 56 | 29.02 |
|  | 6–10 | 50 | 25.91 |
|  | 11–15 | 37 | 19.17 |
|  | 16–20 | 23 | 11.92 |
|  | >20 | 27 | 13.99 |
| Education Level | Bachelor’s degree or below | 64 | 33.16 |
|  | Master’s degree | 98 | 50.78 |
|  | Doctoral degree | 31 | 16.06 |
| Professional Title | None | 10 | 5.18 |
|  | Junior | 61 | 31.61 |
|  | Intermediate | 86 | 44.56 |
|  | Senior | 36 | 18.65 |
| Total |  | 193 | 100.00 |
