# Supplementary material for: Development and psychometric validation of the Physician Occupational Stigma Perception Scale among Chinese physicians
Source: Front Public Health. 2026 Sep 11;14:1912387. doi: 10.3389/fpubh.2026.1912387 (PMC13612318; doi:10.3389/fpubh.2026.1912387)
Supplement: Supplementary file 15 [file Table_9.DOCX]

**Supplementary file 9 Preliminary distributional characteristics of the 12-item POSPS (N=193).**

| **Item** | **Mean** | **SD** | **Min** | **Max** | **Skewness** | **Kurtosis** | **Floor (%)** | **Ceiling (%)** |
| --- | --- | --- | --- | --- | --- | --- | --- | --- |
| OS1 | 3.97 | .910 | 1 | 5 | -0.830 | 0.830 | 2.1 | 31.1 |
| OS2 | 4.06 | .899 | 1 | 5 | -0.688 | 0.076 | 1.0 | 37.8 |
| OS3 | 4.00 | .941 | 1 | 5 | -0.910 | 0.914 | 2.6 | 34.2 |
| OS4 | 4.06 | .801 | 1 | 5 | -0.727 | 0.953 | 1.0 | 31.1 |
| OS5 | 4.10 | .863 | 2 | 5 | -0.241 | -1.498 | 0.0 | 42.0 |
| OS6 | 4.24 | .881 | 1 | 5 | -0.948 | 0.467 | 1.0 | 49.2 |
| OS7 | 3.24 | 1.253 | 1 | 5 | -0.278 | -0.916 | 11.4 | 17.6 |
| OS8 | 3.06 | 1.226 | 1 | 5 | 0.010 | -0.974 | 10.9 | 14.5 |
| OS9 | 2.03 | 1.000 | 1 | 5 | 1.012 | -0.695 | 33.2 | 2.6 |
| OS10 | 3.11 | 1.180 | 1 | 5 | -0.262 | -0.793 | 11.9 | 10.9 |
| OS11 | 3.12 | 1.283 | 1 | 5 | -0.210 | -1.082 | 14.0 | 14.5 |
| OS12 | 3.08 | 1.233 | 1 | 5 | -0.250 | -0.986 | 14.0 | 10.9 |
| Note: Mardia’s multivariate kurtosis = -2.591, c.r. = -0.982. | | | | | | | | |
